# Stronger social bonds do not always predict greater longevity in a gregarious primate

**DOI:** 10.1002/ece3.3781

**Published:** 2018-01-03

**Authors:** Nicole A. Thompson, Marina Cords

**Affiliations:** ^1^ Department of Ecology, Evolution, and Environmental Biology Columbia University New York NY USA; ^2^ New York Consortium in Evolutionary Primatology New York NY USA

**Keywords:** fitness, social partner consistency, social relationships, social ties, survival

## Abstract

In group‐living species, individuals often have preferred affiliative social partners, with whom ties or bonds can confer advantages that correspond with greater fitness. For example, in adult female baboons and juvenile horses, individuals with stronger or more social ties experience greater survival. We used detailed behavioral and life history records to explore the relationship between tie quality and survival in a gregarious monkey (*Cercopithecus mitis stuhlmanni*), while controlling for dominance rank, group size, and life history strategy. We used Cox proportional hazards regressions to model the cumulative (multi‐year) and current (single‐year) relationships of social ties and the hazard of mortality in 83 wild adult females of known age, observed 2–8 years each (437 subject‐years) in eight social groups. The strength of bonds with close partners was associated with increased mortality risk under certain conditions: Females that had strong bonds with close partners that were inconsistent over multiple years had a higher risk of mortality than females adopting any other social strategy. Within a given year, females had a higher risk of death if they were strongly bonded with partners that changed from the previous year versus with partners that remained consistent. Dominance rank, number of adult female groupmates, and age at first reproduction did not predict the risk of death. This study demonstrates that costs and benefits of strong social bonds can be context‐dependent, relating to the consistency of social partners over time.

## 1 INTRODUCTION

Among social animals, individuals commonly have differentiated relationships, or ties, with others. Particularly intriguing are close social bonds, which are characterized by especially high rates of affiliative behavior, including amicable physical contact, and/or particularly close spatial association (Cords & Thompson, 2017). To understand social differentiation from an evolutionary perspective, it is essential to examine its fitness consequences. In humans, for instance, decades of research have shown that the quality and patterning of social ties predict important fitness‐related variables such as disease risk (Uchino, 2006) and mortality (Holt‐Lunstad, Smith, & Layton, 2010). Human studies also suggest that the short‐term effects of social ties accumulate over the long term to influence health outcomes (Uchino, 2006).

More recently, research on nonhuman animals has also linked social relations to direct and indirect measures of fitness, such as reproductive rate (Brent et al., 2013; Farine & Sheldon, 2015; Formica et al., 2011, 2012; Gilby et al., 2013; McDonald, 2007; Schülke, Bhagavatula, Vigilant, & Ostner, 2010; Vander Wal, Festa‐Bianchet, Réale, Coltman, & Pelletier, 2014), offspring survival (Cameron, Setsaas, & Linklater, 2009; Kalbitzer et al., 2017; Silk, Alberts, & Altmann, 2003; Silk et al., 2009; Vander Wal et al., 2014), and longevity (Archie, Tung, Clark, Altmann, & Alberts, 2014; Brent, Ruiz‐Lambides, & Platt, 2017; Fagen & Fagen, 2004; Foster et al., 2012; Lehmann, Majolo, & McFarland, 2016; McFarland et al., 2017; Nuñez, Adelman, & Rubenstein, 2015; Silk et al., 2010b; Stanton & Mann, 2012; Yee, Cavigelli, Delgado, & McClintock, 2008). Among these measures, longevity, or survival, is a particularly important fitness measure in long‐lived mammals, such as primates, that have relatively low reproductive rates (Jones, 2011; Morris et al., 2011). Adult female baboons (*Papio hamadryas ursinus*) in Botswana lived longer if they maintained stronger, more consistent bonds with each other (Silk et al., 2010b). Similarly, female baboons in Kenya (*Papio hamadryas cynocephalus*) lived longer if they were more connected to either adult male or female groupmates (Archie et al., 2014).

Affiliative relationships can influence fitness in several ways (Cords & Thompson, 2017). The general benefits of group living may be amplified by living with especially tolerant and familiar partners. Such partners may work together more efficiently in cooperative hunting (Ruch, Herberstein, & Schneider, 2014), communal care of offspring (Weidt, Lindholm, & Koenig, 2014), or attending to predators (Micheletta et al., 2012). In several species, affiliative partners, often kin, compete more effectively as allies, and alliances help to maintain dominance rank (Chapais, 1995; Mitani, Merriwether, & Zhang, 2000; Schülke et al., 2010) or increase access to mates (Connor, Read, & Wrangham, 2000; Feh, 1999). Affiliative partners may also provide psychosocial support that attenuates prolonged stress responses to events such as infanticide or the loss of close social partners (Engh et al., 2006; Wittig et al., 2008), although links between chronically elevated glucocorticoids and fitness may not be as direct or as prevalent in wild animals as in humans (Beehner & Bergman, 2017). More general integration in social groups, for example, having *more* affiliative partners, may also provide such a buffering effect and protect individuals from environmental risks such as cold temperatures (Lehmann et al., 2016; McFarland & Majolo, 2013; McFarland et al., 2015), enhance access to relevant social and environmental information (Archie, Moss, & Alberts, 2006; Templeton, Reed, Campbell, & Beecher, 2012), and help individuals survive traumatic population‐wide events (Nuñez et al., 2015).

Although most studies emphasize the effects of affiliative and cooperative relations on fitness outcomes, agonistic interactions may also be important. In Barbary macaques, individuals that either received or directed aggression to more partners, and whose aggressive partners were not aggressive toward one another, were more likely to survive a hard winter (Lehmann et al., 2016). Similarly, yellow‐bellied marmots (*Marmota flaviventris*) lived longer if they initiated aggression toward more recipients, although the benefits of aggression here likely derived from its association with social dominance (Lea, Blumstein, Wey, & Martin, 2010).

Indeed, dominance status or rank, derived from agonistic interactions, has a pervasive influence on fitness‐related variables in female mammals, as rank frequently corresponds with priority of access to food (Clutton‐Brock & Huchard, 2013). Dominance rank can predict survival (Pusey, Williams, & Goodall, 1997; Silk et al., 2010b), possibly because high‐ranking individuals are able to access safer microhabitats and avoid predation (van Schaik & Van Noordwijk, 1986), better access nutrients (Foerster, Cords, & Monfort, 2011), and avoid harassment during development (Silk, Samuels, & Rodman, 1981). In our study species, the blue monkey, rank has no effect on conception probability (Roberts & Cords, 2013), but higher‐ranking females had lower baseline glucocorticoid levels during an energetically challenging period when lactation overlapped with low food availability (Foerster et al., 2011).

Variables other than the quality of social ties and rank can also influence survival in group‐living animals. Large group size may enhance survival by providing benefits similar to those of maintaining particularly affiliative relationships, such as more effective vigilance for predators (Elgar, 1989; Lehtonen & Jaatinen, 2016; Roberts, 1996; van Schaik & Van Noordwijk, 1986), defense of young offspring (Grinnell & McComb, 1996; Wolff & Peterson, 1998), or defense of feeding territories (Radford & du Plessis, 2004; Roth & Cords, 2016). Nevertheless, living in larger groups may also exact costs by increasing within‐group competition for food (Roberts & Cords, 2013; VanderWaal, Mosser, & Packer, 2009), or by increasing the risk of male takeovers and subsequent infanticide (Steenbeek & van Schaik, 2001). In some cases, the way the cost–benefit balance changes in larger social groups means that intermediate group sizes are optimal for individual fitness (Markham, Gesquiere, Alberts, & Altmann, 2015; Roberts & Cords, 2013). Finally, at a basic life history level, individuals may trade off energetic investment in somatic growth and maintenance (survival) for reproduction (Descamps, Boutin, Berteaux, & Gaillard, 2006; Hamel et al., 2010).

In this study, we used survival analyses to examine the link between affiliative social ties and lifespan of wild adult female blue monkeys, while also controlling for the influence of other socio‐demographic factors and a potential life history trade‐off. We examined both the cumulative (multi‐year) and current (annual) effect of social experience on survival, using fixed‐effect and time‐dependent Cox models to test for each, respectively. We focused mainly on affiliative relations because agonistic interactions occur at low rates in this species (Klass & Cords, 2015).

Although blue monkeys differ from other cercopithecines in multiple ways, we expected to confirm patterns documented in certain macaques (Lehmann et al., 2016) and baboons (Archie et al., 2014; Silk et al., 2010b), namely, that more or higher‐quality social ties, either cumulatively over multiple years or in one's current environment, correspond with higher survival. Unlike these other species, blue monkeys are highly arboreal, live in a less seasonal (rainforest) environment, seem to experience relatively strong feeding competition between groups but relatively weak competition within groups (Cords, 2007; Klass & Cords, 2015), and live in groups with a single male. Despite these differences, we hypothesized that strong and stable female–female affiliative ties would enhance longevity in female blue monkeys because, like other cercopithecines, they have a female‐philopatric and matrilineally structured society in which grooming is the most obvious form of affiliation. We combine long‐term life history data with detailed behavioral observations from an 8‐year period on a wild population. We predicted specifically that females that maintained consistent and strong bonds with their closest partners would have a survival advantage relative to females with weak and inconsistent bonds, and that the benefits of strong and consistent bonds would be more pronounced over the long term.

## 2 MATERIALS AND METHODS

### 2.1 Study site and population

The study population inhabits the Isecheno area of Kakamega Forest in western Kenya (0°19′N, 34°52′E; elevation 1580 m, mean annual rainfall 1997–2011 1942 mm; Mitchell 2009). Natural predators occur here, including the African crowned eagle (*Stephanoaeutus coronatus*) and Gaboon viper (*Bitis gabonica*, Gaynor & Cords, 2012). The ca. 2 km^2^ study area supports a high density of blue monkeys, with approximately 192 individuals/km^2^ in old secondary forest and fewer in mixed indigenous plantations (Fashing et al., 2012). Between‐group territorial disputes are common, occurring about every other day (Cords, 2007). Blue monkey groups usually comprise a single adult male, multiple adult philopatric females, and their young. This population has been monitored since 1979, and all study group members were identifiable based on natural physical variation (Cords, 2012).

During the period of data collection for this study (2006–2014), we observed 83 adult (parous) female subjects in eight study groups, four of which resulted from two fission events that occurred in 2008 and 2009 (Figure 1 in Klass & Cords, 2015). In addition, in one group, three juveniles of unknown provenance joined the group in 2010 and became adult subjects in our analysis.

**Figure 1:**
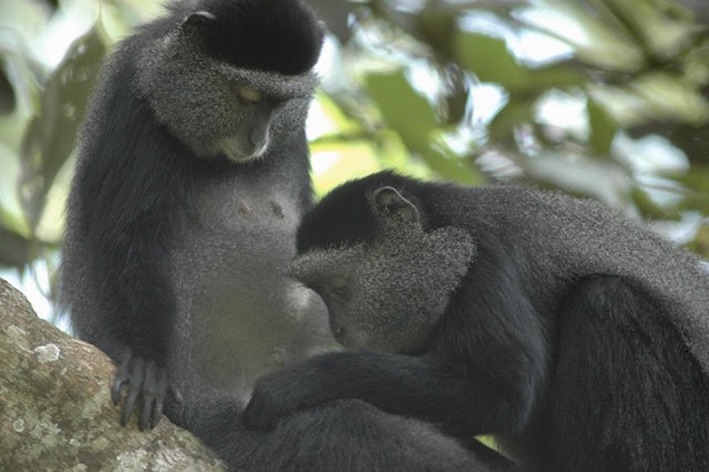
Two adult female blue monkeys grooming

Long‐term monitoring of demographic events (Cords, 2012) allowed us to specify birth and death dates; females alive at the end of the study (*N* = 63) were right‐censored. For 12 of the oldest adult females and three who had immigrated as juveniles, we estimated birthdates based on changes in juvenile body size (average precision ± 0.75 years, range: 0.08–2). We inferred most deaths based on permanent disappearances as observers rarely found carcasses of missing animals. Female dispersal from the natal group is extremely rare in this species (two possible events in 111 group‐years of monitoring, although these may have been small group fusions, author MC pers. obs.), and several females that disappeared either left behind young offspring (<2 years) or were in poor physical condition prior to disappearance. Cause of death was seldom known, but most deaths probably resulted from intrinsic factors or predation.

### 2.2 Behavioral data collection

Detailed records of adult female social behavior, based on focal animal sampling, were available from October 2006. A team of observers trained by and including MC conducted 30 min samples on subjects approximately once every 3 days throughout the year (mean ± *SD* observation hours per subject‐year = 67 ± 20, *N* = 437). On a given day, observers chose focal subjects so as to even out the sampling rate across individuals and to balance observation time across the hours of the day. Subjects were observed for 2–8 years each (mean = 5.26). We considered a subject to be present in a given observation year if she was a subject for >10% of the year (69 of 467 subject‐years < full year, mean days observed if < full year = 310).

During focal samples, observers made instantaneous records at 1‐min intervals of subject activity (including grooming, resting, feeding, and moving) and the identity of all “neighbors” within 1 m whenever the subject was grooming or resting, or within 7 m if she was feeding. Because of the dense vegetation, subjects sometimes went out of sight. If observers relocated the subject within 15 min, they continued the sample until they achieved 30 min of observation; otherwise, the sample was terminated. Samples lasting <20 min were discarded.

Data on agonism, used to calculate dominance rank, came from focal and ad libitum observations (Klass & Cords, 2015). Observers recorded winners and losers in all decided agonistic interactions (in which one and only one opponent showed submission).

### 2.3 Data analysis

#### 2.3.1 Social predictors of survival

In many primates, mothers of young infants attract extra social attention. To measure social interaction that was not driven by short‐term attraction to infants (Henzi & Barrett, 2002), we removed observation records when a subject or her partner had an infant <100 days old. Infant blue monkeys begin to spend a substantial time away from their mothers at this age (Förster & Cords, 2005).

To see how the quality of social bonds affected survival, we first calculated bond strength for a given subject and all her adult female social partners in a given year. For this, we used an annual dyadic sociality index (DSI, Silk, Cheney, & Seyfarth, 2013) based on grooming and time spent resting in proximity (1 m), each expressed as a proportion of total dyad observation time (which was the sum of time observed for each dyad member as a subject). Grooming and resting within 1 m are two measures of affiliation known to be strongly biased toward maternal kin (Cords & Nikitopoulos, 2015). Matrices of dyadic proportions of time spent grooming or resting in proximity were correlated in 28 of 43 group‐years, so their combination in a composite index seemed justified (electronic supplementary material, Table S1). We calculated the index as follows:

$$\mathrm{DSI}=\frac{1}{2}\left(\frac{G_{\mathrm{ij}}}{G_{\mathrm{med}}}\right)+\frac{1}{4}\left(\frac{R_{\mathrm{ij}}}{R_{\mathrm{med}}}+\frac{R_{\mathrm{ji}}}{\;R_{\mathrm{med}}}\right)$$

where *G*_*ij*_ represents the proportion of time that the dyad members spent grooming, *R*_*ij*_ and *R*_*ji*_ represent the proportion of time each dyad member *i* and *j*, as focal subjects, spent resting within 1 m of the other (without grooming or feeding), and *G*_med_ and *R*_med_ are the median values of all within‐group dyads across social groups in the same year. We divided the resting association data for a given dyad into two equal components based on focal subject identity to account for the fact that resting proximity was not symmetrical within the dyad (i.e., a resting focal subject might have a neighbor who was feeding, when observers scored proximity partners within a larger 7‐m distance). A DSI of 1 would represent a typical dyad, while values >1 represent a dyad with stronger than median social ties.

To characterize each subject's bondedness over multiple years, we first averaged DSIs with her top three partners in a given year and then averaged over her annual values. We chose to average the DSIs of a female's top three partners for two reasons. First, across several species of social mammals, individuals tend to associate with decreasing intensity across social partners in tiers that scale by a multiple 3–3.15 (e.g., tier_1_ = individual, tier_2_ = grooming clique, Zhou, Sornette, Hill, & Dunbar, 2005; Hill, Bentley, & Dunbar, 2008). Second, averaging over top three partners allowed us to compare results with previous landmark studies on closely related primates (Silk et al., 2003, 2009). Nevertheless, to assess whether ties with top three partners specifically were meaningful, we also explored the influence of DSIs averaged over the top six closest partners.

To measure partner consistency over multiple years, we first identified those individuals among the top three partners that were “consistent,” and then asked what proportion of a female's top three partner “slots,” across the years in which she was observed, were occupied by such consistent partners. We considered a top partner in a given year to be consistent if her DSI continued to place her in the top three positions at least once in the next two years (Silk et al., 2009). In a subject's second to last year of observation, we counted a top partner as consistent if she was among the top three in the next year only. We then determined what fraction of a female's top three “slots,” summed across years, were occupied by a consistent partner. The number of “slots” was 3Y‐3, where Y is the total number of observation years; we subtracted 3 because we could not assess consistency status of partners in the last year. This fraction varied from 0 (low consistency) to 1 (high consistency). Partner consistency was not related to number of years observed. To correspond with our alternative measure of bond strength with top six partners, we also calculated consistency in top six partners over time. During a few years, some subjects lived in groups with fewer than six or even three adult female groupmates. These comprised 7% and 1% of 437 subject‐years, respectively.

To measure current partner consistency (i.e., within a given year), we counted the proportion of a female's top three or six partners that were present among her top partners at least once in the previous 2 years. For a female's second year of observation, we counted the proportion of top partners present from the previous year of observation alone. As the consistency of partners in a female's first year of observation could not be measured, annual partner consistencies were calculated for 354 of 437 subject‐years. We calculated annual partner consistency retrospectively because we considered current (not future) partner stability relative to previous years to be most relevant to survival in the same year. Although prospective and retrospective measures of multi‐year partner consistency do not differ greatly, measuring multi‐year consistency prospectively assesses whether a female invested in partners that then remained consistent, and perhaps whether current partner choices would pay off over time.

Similar to a previous study (Silk et al., 2010b), we wished to condense bond strength and partner consistency into a single measure of relationship quality. Multi‐year bond strength and partner consistency were correlated (*N* = 83, *r* = .23, *p* = .03); however, they were not correlated so closely as to load on a single principal component (Table S2). The relative independence of these variables led us to categorize females in one of the four classes of above (+) and below (−) population mean bond strength and partner consistency, where class 1 = ‐ strength & ‐ consistency (*N* = 29), 2 = ‐ strength & + consistency (*N* = 20), 3 = + strength & ‐ consistency (*N* = 11), and 4 = + strength & + consistency (*N* = 23). We also categorized females using measures of bond strength and partner consistency among her top six partners (classes 1 −/− *N* = 39, 2 −/+ *N* = 11 +/− *N* = 16, 3 +/+ *N* = 17). To better understand if females were forced into particular multi‐year classes because of the deaths of social partners, we calculated how often deaths were responsible for partner changes and whether the proportion of death‐induced changes differed among females by class.

To characterize current relationship quality, we again created an index in which females were placed in four classes of above or below average annual bond strength and above or below average annual partner consistency. These classes were populated relative to one another in a similar way as multi‐year strength–consistency classes (top three class 1 −/− *N* = 112 subject‐years, 2 −/+ *N* = 94, 3 +/− *N* = 60, 4 +/+ *N* = 88; top six class 1 *N* = 150, 2 *N* = 65, 3 *N* = 84, 4 *N* = 55).

We calculated dominance ranks from records of decided agonistic interactions among adult females using the I&SI method as implemented in DomiCalc (Schmid & de Vries, 2013). We expressed ranks as the proportion of adult female groupmates a female outranked in each year. For multi‐year analyses, we averaged subjects' annual ranks over all years in which she was observed.

#### 2.3.2 Demographic and environmental predictors of survival

Long‐term records allowed us to specify females' age at first birth and the number of adult female groupmates (Cords, 2012). Average number of adult female groupmates closely approximated a female's average number of adult female grooming partners (*N* = 83, *r* = .8, *p* < .001), as annual grooming networks among female groupmates were saturated or nearly so. We therefore included only number of adult female groupmates, and not number of grooming partners (which would provide no additional information), as a predictor of survival.

### 2.4 Statistical analysis

#### 2.4.1 Repeatability of social behavior

To assess the validity of averaging bond strength, dominance rank, and adult female groupmates over time to derive single‐, multi‐year values for each subject, we tested the repeatability of interindividual differences in each predictor by calculating the intraclass correlation coefficient from a linear mixed effects model (function rpt in R package “rptR,” Nakagawa & Schielzeth, 2010). The model calculates the proportion of total variance among all annual measures of a given variable that is attributed to variation between individuals, which are modeled as random effects, while controlling for variance explained by other social or environmental variables, modeled as fixed effects.

#### 2.4.2 Survival analysis

We used both fixed‐time and time‐dependent Cox proportional hazards regressions (function coxph in R package “survival,” Therneau & Grambsch, 2000; Therneau, 2015) to assess the cumulative (fixed‐time) and current (time‐dependent) influence of social tie quality (bond strength and consistency class with top partners), dominance relationships (rank), group size (number of adult female groupmates), and life history strategy (age at first birth) on a subject's instantaneous risk of death. Survival intervals were left‐truncated at a subject's age when focal animal sampling began in October 2006 (if she was an adult then) or at the subject's age at first birth (if she became an adult later). In all models, we used standardized covariates (Schielzeth, 2010).

We considered a predictor to influence the hazard if the 95% confidence interval of its parameter estimate did not include zero (Nakagawa & Cuthill, 2007). Because bond strength‐partner consistency classifications were based on dyadic data, we additionally examined their influence on the hazard according to permutation tests, comparing observed effects of strength–consistency class to a null model based on 1000 random node permutations of annual DSI matrices (Croft, Madden, Franks, & James, 2011; Farine, 2017). We chose node permutations to test the null hypothesis based on the possibility that females could maintain any position within a social group's annual network. Because of a lack of consensus in the literature as to whether permutation tests are appropriate when relational social measures are *independent* variables (Anderson & Legendre, 1999; Dekker, Krackhardt, & Snijders, 2007; Lehmann et al., 2016; VanderWaal, Atwill, Hooper, Buckle, & McCowan, 2013), we compared 95% CIs and permutation to assess their agreement, and if significance based on 95% CIs disagreed with permutation tests, we gave prominence to 95% CI results.

For fixed‐time covariate Cox models, we averaged annual measures across all years in which the subject was observed, effectively testing the cumulative effect of multiple years of social conditions on survival. Survival models have sufficient power when each variable corresponds with 5—10 events (Vittinghoff & McCulloch, 2007). Given our sample size of 20 deaths in 83 females, we created two models with three predictors each. Both tested the influence of social ties (strength–consistency class) on survival and controlled for the effects of a potential life history trade‐off (age at first reproduction). One model included adult female dominance rank and the other included number of adult female groupmates as measures of social competition. We report model‐averaged parameters of strength–consistency class and age at first birth, as their effects did not differ qualitatively between the model including dominance rank and the model including number of adult female groupmates (“modavg” function in R package “AICcModavg,” Mazerolle, 2016). To assess the possibility that female survival was highest in groups of intermediate size, we also explored two additional models testing the significance of a quadratic relationship between survival and number of adult female groupmates, alongside subjects' strength–consistency class for top three and top six partners and her age at first reproduction. We tested that all models, with either dominance rank or number of female groupmates, met the proportional hazards assumption by assessing the correlation of their Schoenfeld residuals to transformed time (cox.zph function in R package “survival,” Therneau & Grambsch, 2000; Therneau, 2015).

In the time‐dependent covariate model, variables per subject‐year appeared as separate observations to predict a female's risk of death in the same year. This approach effectively tested the time‐dependent relationship between current social conditions and survival. We constructed separate models for measures with top three and six partners, which included annual values of strength–consistency class, dominance rank, and number of adult female groupmates.

## 3 RESULTS

Averaging annual measures of social predictor variables appeared to be a valid approach, as each predictor showed repeatable interindividual differences (electronic supplementary material, Table S3). Nevertheless, as the lower confidence limit of bond strength's repeatability statistic was close to zero, a female's bond strength did appear to vary from year to year. Such intraindividual variation supported our analysis of time‐dependent predictors.

Across 83 females, the average female's bond strength with her top three partners was 7.6 ± 2.5, that is, 7.6 times greater than the median bond strength between any two adult coresident females, and 51 ± 18% of the top three partner identities remained consistent (as per definition) during her observation period (electronic supplementary material, Table S4). Females lived with an average of 13.6 ± 4.2 adult female groupmates (range 3–21).

**Figure 2:**
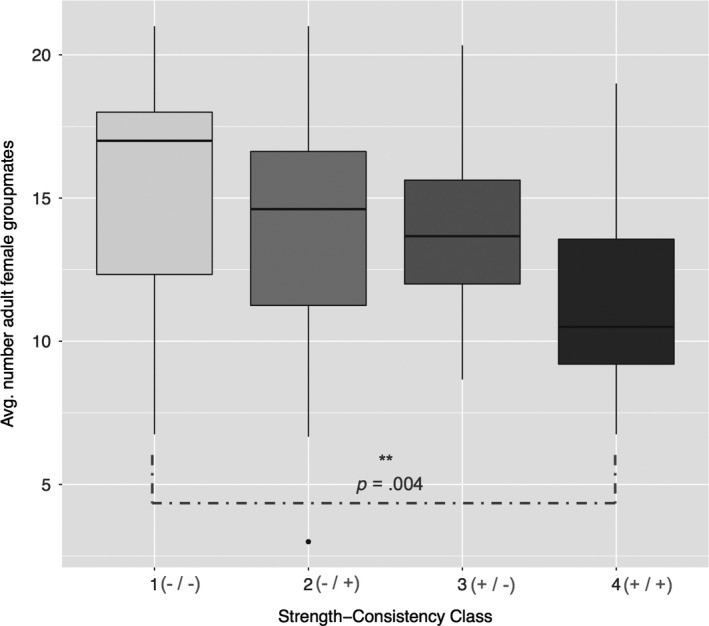
Number of adult female groupmates per female (median and IQR,* N* = 83) by multi‐year strength–consistency class of top three partners. Group sizes were significantly different for females in class 1 versus 4 (see text)

Measures of bond strength and partner consistency with top three partners were each highly correlated with their corresponding measure including top six partners (Table S4). Each multi‐year measure decreased as group size increased, such that females had weaker and less consistent close partners in larger social groups (Table S4). Indeed, average number of adult female groupmates varied by strength–consistency class with top three partners, such that females that had strong and consistent partners (class 4, +/+) lived on average in smaller groups than females that had weak and inconsistent bond partners (class 1, −/−; Tukey's HSD, difference classes 4–1 = −3.89, 95% range = −6.82 to −0.96, *p* = .004; Figure 2). Number of adult female groupmates did not differ between any other classes of relationship quality.

Deaths accounted for 13.8% of the average female's changes in top three partners (*N* = 82 females with changes in top three) and 19.6% of changes among her top six partners (*N* = 80 females with changes in top six). Proportion of partner changes resulting from death did not differ among females according to their multi‐year, top three strength–consistency classes (anova *F*_3,78 _= 1.99, *p* = .12). However, females that were weakly bonded to a consistent set of top six partners (class 2) experienced a higher proportion of death‐related partner changes than females with weak and inconsistent (class 1; *N* = 80, Tukey's HSD, difference classes 2–1 = 0.29, range = 0.10–0.48, *p* = .001) and strong and inconsistent top six partners (class 3, difference classes 3–2 = −0.31, range = −0.52 to −0.094, *p* = .002). It is likely that females with few partner changes consequently had a higher proportion of changes resulting from deaths.

### 3.1 Influences on risk of death

**Figure 3:**
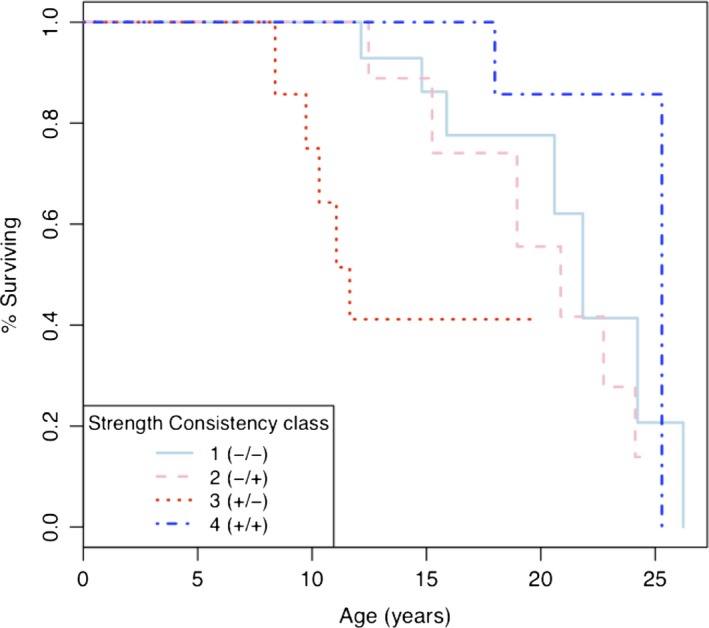
Survival curve of subjects in four multi‐year bond strength–partner consistency classes (with top three partners): class 1 below average bond strength and below average partner consistency (light blue, solid line). Class 2 below average bond strength and above average consistency (pink, dashed line). Class 3 above average bond strength and below average consistency (red, small dotted line). Class 4 above average strength and above average consistency (dark blue, dashed and dotted line)

Risk of death among adult females varied according to multi‐year relationship quality with their top three partners (Table 1, Figure 3). Females that had above average strength bonds with less than average consistency in partners (class 3, +/−) had a higher risk of death than females in all other strength–consistency classes, according to both 95% CI's of parameter estimates and permutation tests (Table 1, Figure 3). The difference in the hazards of classes 1 (−/−), 2 (−/+), and 4 (+/+) did not reach significance according to 95% CIs (Tables S5 and S6). Yet according to permutation tests, the hazard ratio of females with weak and inconsistent bonds (class 1, −/−) versus females with strong and consistent bonds (class 4, +/+) was significantly higher than expected by chance. There were no differences in risk between intermediate‐risk classes 1 and 2 according to either 95% CIs or permutation tests.

**Table 1:** Influence of (standardized) fixed‐time predictors on risk of death. *N* = 83 females, 20 deaths

Predictor class	Predictor of hazard	Factor level	ß	95% CI	Hazard ratio	Proportion of permutation coefficients < observed
Social ties	Strength–consistency class (reference class: 3, +/−)	1 (−/−)	−2.1^a^	−3.53, −0.62^b^	0.13	0.001^c^, 0.02^d^
		2 (−/+)	−1.5^a^	−2.91, −0.13^b^	0.22	0.01^c^,^d^
		4 (+/+)	−3.0^a^	−4.83, −1.2^b^	0.05	0^c^,^d^
Competition	Dominance rank	n/a	0.06	0.52, 0.64	1.06	n/a
	Number of adult female groupmates	n/a	−0.23	−0.77, 0.31	0.80	n/a
Life history	Age at first birth	n/a	−0.52^a^	−1.12, 0.07	0.59	n/a

a Model‐averaged coefficient.

b 95% CI does not include zero.

c From model 1: including dominance rank as competition variable.

d From model 2: including number of adult female groupmates as competition variable.

When we considered a females' multi‐year relationship quality with top six partners, there were no significant contrasts in the hazards among strength–consistency classes according to 95% CIs. According to permutation tests, however, classes 1 (−/−) and 3 (+/−) had similar hazards that were significantly higher than classes 2 (−/+) and 4 (+/+, Tables S7–S9). Permutation tests therefore emphasized a positive influence on survival of consistency among top six partners.

The significant effects according to permutation tests are potentially false positives, which may arise as a result of breaking ancillarity (Anderson & Legendre, 1999; Dekker et al., 2007). Permuting values of a predictor variable in a multiple regression breaks ancillarity if there is any collinearity among predictor variables, as it removes any relationships between them. Indeed, there were several unavoidable correlations among bond strength, partner consistency, rank, and group size (Table S4), although collinearity among them in linear regression Cox models was not problematically high (max VIF all models, excluding model with quadratic term = 1.23). These contrasts in results according to parametric versus permutation‐based null hypotheses may contribute further to discussion in the ecological literature about the suitability of permuting an independent social variable when estimating its partial regression coefficient.

Neither multi‐year dominance rank's nor group size's influence on survival reached significance (Table 1, fixed‐time models). Group size also did not demonstrate a quadratic relationship with survival (Tables S10 and S11). Age at first reproduction did approach significance in the expected direction, such that later ages at maturity would increase longevity (Table 1). All models including either dominance rank or number of adult female groupmates as a competition variable did not depart from proportional hazards (all global *p* > .10).

Time‐dependent covariate models revealed patterns similar to those of fixed‐time models. Strong bonds with few top three partners from the previous two years (class 3: + strength/‐ consistency) were associated with a higher risk of death than having strong bonds with consistent partners from previous years (class 4 +/+, Cox proportional hazards, *N* = 354, ß _class 3 vs. 4_ = −1.52, hazard ratio = 0.22, 95% CI = −3.03 to −0.01; electronic supplementary material, Tables S12 and S13, Figures S2 and S3). However, no other comparison between annual strength–consistency classes was more or less hazardous than the other. Fewer between‐class comparisons reached significance in time‐dependent models and the lower 95% confidence limit of the coefficient of class 3 versus 4 was very near zero, suggesting that annual strength–consistency class had a weaker effect on survival than strength–consistency classes based on multiple years. The effects of annual dominance rank and annual group size on survival did not reach significance in models of either top three or top six partners. strength–consistency class with top six partners also did not significantly influence survival in a time‐dependent way.

## 4 DISCUSSION

### 4.1 Influences on the risk of death

The quality of a female's social ties with her closest three partners, assessed both over multiple and single years, predicted survival in adult female blue monkeys. Specifically, over multiple years of observation, a female's risk of death was highest if she had strong bonds with a set of top three partners that was inconsistent from year to year. Females that were weakly bonded over multiple years, with either consistent or inconsistent partners, or strongly bonded with consistent partners all had similarly lower risks of death than females with strong and inconsistent partners. Similarly, a female that was strongly bonded in a given year with partners that had changed from previous years had a higher risk of dying in that same year than females that were strongly bonded with consistent partners. Neither multi‐year nor current annual relationship quality with her closest six partners influenced female survival.

We did not find clear evidence of a life history trade‐off between survival and reproduction (Table 1, fixed‐time analysis). If early investment in reproduction does compromise somatic maintenance, these results emphasize the greater influence of cumulative social relationship quality versus physical condition on survival in blue monkeys. Similarly in bighorn ewes, social ties had a stronger effect than body mass on survival (Vander Wal et al., 2014). We also found no evidence of an effect of dominance rank and group size on survival, either over multiple years or in a particular year.

Maintaining strong bonds when partners are inconsistent from year to year (highest risk multi‐year strategy, Table 1 Figure 3) may represent an investment that outweighs the return, that is, females invest in partners that are too inconsistent to reciprocate or cooperate as allies. In general, consistent partners help to create a stable social environment, and the loss of important partners can elicit a stress response (Engh et al., 2006). Affiliative partners that persist over time may also promote reciprocal grooming (Taborsky, 2013) or provide coalitionary or affiliative support on a subject's behalf during or after an aggressive encounter (Silk et al., 2010a). More passively, consistent partners may tolerate a subject's presence during feeding (Marshall, Carter, Coulson, Rowcliffe, & Cowlishaw, 2012). The benefits of consistent partners are presumably amplified when partners affiliate more intensely (Silk et al., 2010b). The fact that strong bonds over multiple years actually decreased survival when partners were inconsistent, rather than having a neutral influence, suggests that maintaining strong bonds may be costly to blue monkey females.

The effects of current relationship quality on immediate survival (in a time‐dependent model) also suggested that strong bonds were costly. Maintaining currently strong bonds with few close partners from previous years was riskier than being strongly bonded with many previously close partners. This finding emphasizes that if a female maintains strong bonds, she should do so with partners that are relatively consistent. It also suggests that females may not only lose the return on investment in strong bonds with future inconsistent partners, but they may also pay a cost when associating most frequently with “new” partners.

The stronger effect of multi‐ versus single‐year strength–consistency class on survival further suggests that the cost of maintaining strong bonds with inconsistent partners is cumulative. Only after several years of investment in partners that change from year to year are females disadvantaged relative to females maintaining *any* other strategy of bondedness with close partners (i.e., even being weakly bonded with inconsistent partners or weakly bonded with consistent partners is a better strategy).

If maintaining bonds is costly, being weakly bonded may actually be a beneficial strategy over multiple years. In fact, maintaining weak bonds with either consistent or inconsistent partners were both lower risk than maintaining strong bonds with inconsistent partners over time. Females that are weakly bonded spend less time on partners, and so perhaps never pay the time, energy, or exposure cost of maintaining strong bonds.

Demographic constraints such as group size, but not partner deaths, may underlie the uncoupling of bond strength and partner consistency in blue monkeys. Females with the most hazardous combinations of strong bonds with inconsistent partners over multiple years tended to live in groups of intermediate size. Meanwhile, females that lived with relatively more or fewer females had weak and inconsistent or strong and consistent partners, respectively. While the greater availability of different social partners may understandably decrease the consistency of close partners from year to year, extreme (vs. intermediate) group sizes may facilitate females' beneficial tendency to maintain either strong bonds with consistent or weak bonds with inconsistent partners. Although the riskiest social strategy tended to occur in groups of intermediate size, number of adult female groupmates did not appear to have a quadratic relationship with female survival.

### 4.2 Comparison with other social species

This study is the first survival analysis to examine how social connections influence longevity in an arboreal primate (Archie et al., 2014; Brent et al., 2017; Foster et al., 2012; McFarland et al., 2017; Nuñez et al., 2015; Silk et al., 2009, 2010b; Stanton & Mann, 2012; Yee et al., 2008) and to compare the cumulative versus current effects of relationship quality. Results both concur with and differ from these and other previous studies of how social relations influence other direct fitness measures (Brent et al., 2013; Cameron et al., 2009; Gilby et al., 2013; Kalbitzer et al., 2017; McDonald, 2007; Schülke et al., 2010; Silk et al., 2003; Vander Wal et al., 2014).

Similar to all the above studies, we found that greater sociality in the form of stronger top bonds can indeed correspond with higher survival in blue monkey females. However, in contrast to several previous studies, strong bonds actually correspond with lower survival in certain situations (i.e., when bond partners change from year to year), suggesting that maintaining bonds is costly. Indeed, over multiple years, females that were weakly bonded had a lower risk of death than females that were strongly bonded to partners that were inconsistent over time.

Blue monkey females seem to receive a return on their social investment only if bond partners are relatively consistent. Somewhat similarly, affiliation appears to be costly to female marmots because strongly affiliating females produced fewer offspring (although the causal relationship between affiliation and reproduction was not clear, Wey & Blumstein, 2012) and are more likely to die during hibernation (Yang, Maldonado‐Chaparro, & Blumstein, 2016). Also similarly, in white‐faced capuchin, the costs and benefits of females' strong bonds depend on male behavior (Kalbitzer et al., 2017). The offspring of strongly bonded females was more likely to fall victim to infanticide during alpha male replacements, but during periods of alpha male stability, they were more likely to survive. As the strongest bonds of blue monkey females are not necessarily with a consistent set of partners (unlike in baboons, Silk, Alberts, & Altmann, 2006; Silk, Altmann, & Alberts, 2006; Silk et al., 2010b), females may actually benefit by saving the time and energy spent on cultivating strong bonds. Those savings and their benefits remain to be quantified on a mechanistic level. Because within‐group agonism and alliances are rare in blue monkeys (Klass & Cords, 2015), the function of their social bonds in general may be to maintain group cohesiveness rather than orchestrate competitive power relations within groups. Group‐wide cohesion may not require particularly strong bonds.

## 5 CONCLUSIONS

We found that stronger bonds do not necessarily increase survival in females in a matrilocal, gregarious species. In adult female blue monkeys, stronger bonds with close social partners decreased survival when close partners were inconsistent over time. When strong bonds were consistent over multiple years, survival was high relative to all other strategies. It appears, then, that strong bonds may be costly to adult females and are a productive “investment” only in situations where their benefit outweighs their cost. Other longitudinal, individual‐based studies that examine the influence of social ties on fitness may find it useful to incorporate variables that capture variation in the potential costs and benefits of social ties.

In general, studies such as ours that find a correlation between the quality of social ties and survival in animals are only a first step in understanding the actual mechanisms by which social ties influence fitness. Although it is a regular challenge of long‐term field studies to obtain high‐resolution data on individuals' physiological status and social interactions simultaneously, future studies should aim to resolve the three‐part connection between social ties, physiological status, and fitness outcomes whenever possible. Consideration of social measures and timescales relevant to study species will help us to understand how social ties influence fitness.

## COMPETING INTEREST

The authors have no competing interests.

## AUTHOR CONTRIBUTION

NT and MC conceived the ideas and designed methods; MC collected the data; NT and MC analyzed the data; NT led manuscript writing. Both authors contributed critically to the drafts and gave final approval for publication.
