# Supplementary material for: Stronger social bonds do not always predict greater longevity in a gregarious primate
Source: Ecol Evol. 2018 Jan 3;8(3):1604–14. doi: 10.1002/ece3.3781 (PMC5792528; doi:10.1002/ece3.3781)
Supplement: Supplementary file 5 [file ECE3-8-1604-s005.docx]

**Multi-year strength-consistency class with top 3 partners: comparisons between different reference classes:**

Table S5. Average influence of multi-year strength-consistency class (top 3 partners) in fixed-time survival models, where reference class = 4, strong and consistent bonds. N=83 females, 20 deaths.

| Predictor of hazard | Factor level | ß | 95% CI | | Hazard Ratio | Proportion of permutation coefficients < observed | Proportion of permutation coefficients > observed |
| --- | --- | --- | --- | --- | --- | --- | --- |
| Strength – Consistency Class  (reference class:  4, + / +  lowest risk) | 1 (- / -) | 0.93 † | -0.72, 2.6 | 2.55 | | **1^1^, 0.999^2^** | **0^1^, 0.001^2^** |
|  | 2 (- /+) | 1.49 † | -0.2, 3.18 | 4.45 | | 0.958^1^, 0.961^2^ | 0.042^1^, 0.039^2^ |
|  | 3 (+/ -) | 3.01 † | **1.2, 4.83 *** | 20.39 | | **1^1,2^** | **0^1,2^** |

† Model averaged coefficient

* 95% CI does not include zero

^1^From model 1: including dominance rank as competition variable.

^2^From model 2: including number of adult female groupmates as competition variable.

Table S6. Average influence of multi-year strength-consistency class (top 3 partners) in fixed-time survival models, where reference class = 1, weak and inconsistent bonds. N=83 females, 20 deaths.

| Predictor of hazard | Factor level | ß | 95% CI | | Hazard Ratio | Proportion of permutation coefficients < observed | Proportion of permutation coefficients > observed |
| --- | --- | --- | --- | --- | --- | --- | --- |
| Strength – Consistency Class  (reference class:  1, - / -  second to lowest risk) | 2 (- / +) | 0.56 † | -0.73, 1.84 | 1.74 | | 0.069^1^, 0.065^2^ | 0.931^1^, 0.935^2^ |
|  | 3 (+/ -) | 2.1 † | **0.62, 3.53 *** | 8.0 | | **0.999^1^, 0.984^2^** | **0.001^1^, 0.016^2^** |
|  | 4 (+/+) | -0.93 † | -2.6, 0.72 | 0.39 | | **0^1^, 0.01^2^** | **1^1^, 0.99^2^** |

† Model averaged coefficient

* 95% CI does not include zero

^1^From model 1: including dominance rank as competition variable.

^2^From model 2: including number of adult female groupmates as competition variable.
