# Supplementary material for: Stronger social bonds do not always predict greater longevity in a gregarious primate
Source: Ecol Evol. 2018 Jan 3;8(3):1604–14. doi: 10.1002/ece3.3781 (PMC5792528; doi:10.1002/ece3.3781)
Supplement: Supplementary file 6 [file ECE3-8-1604-s006.docx]

**Multi-year strength-consistency class with top 6 partners results:**

**
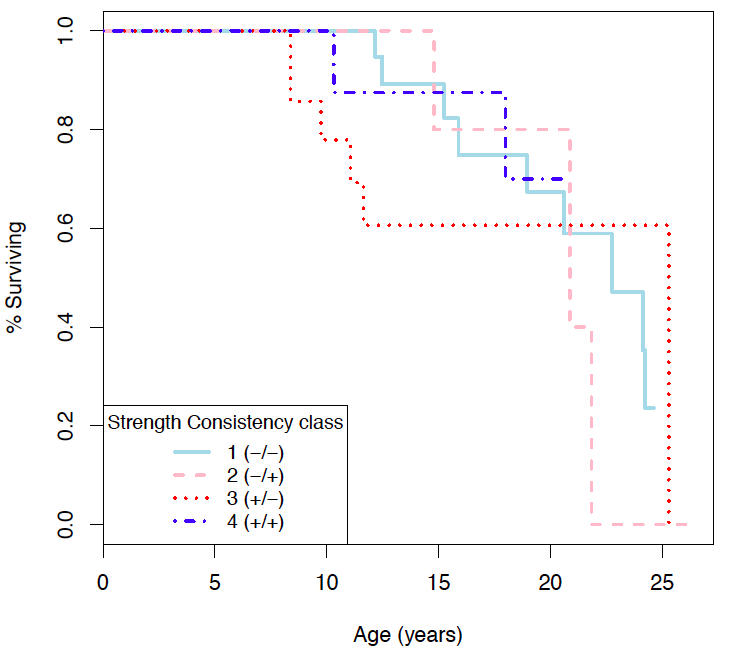
**

Figure S1. Survival curve of subjects falling into a given multi-year bond strength – partner consistency (with top 6 partners) class. Class 1) below average bond strength and below average partner consistency (light blue, solid line). Class 2) below average bond strength and above average consistency (pink, dashed line). Class 3) above average bond strength and below average consistency (red, small dotted line). Class 4) above average strength and above average consistency (dark blue, dashed and dotted line). Female survival did not significantly vary by class according to coefficients’ 95% CI.

Table S7. Average influence of multi-year strength-consistency class (top 6 partners) in fixed-time survival models, where reference class = 3, strong and inconsistent bonds (highest risk class). N=83 females, 20 deaths.

|  | Predictor of hazard | Factor level | ß | 95% CI | | Hazard Ratio | Proportion of permutation coefficients < observed | Proportion of permutation coefficients > observed |
| --- | --- | --- | --- | --- | --- | --- | --- | --- |
| Social ties | Strength – Consistency Class  (reference class:  3, + / -  highest risk) | 1 (- / -) | -0.39† | -1.56, 0.79 | 0.68 | | 0.517^1^, 0.625^2^ | 0.549^1^, 0.409^2^ |
|  |  | 2 (- /+) | -0.47† | -2.07, 1.13 | 0.62 | | **0.005^1^, 0.002^2^** | **0.995, 0.998^1,2^** |
|  |  | 4 (+/ +) | -0.82† | -2.68, 1.05 | 0.44 | | **0.011^1^, 0.01^2^** | **0.989^1^, 0.99^2^** |
| Competition | Dominance rank  Number adult female groupmates | n/a | 0.13 | -0.37, 0.62 | 1.14 | | n/a | n/a |
|  |  | n/a | -0.44 | -0.97, 0.29 | 0.64 | | n/a | n/a |
| Life history | Age at first birth | n/a | -0.46 | -1.01, 0.09 | 0.63 | | n/a | n/a |

† Model averaged coefficient

* 95% CI does not include zero

^1^From model 1: including dominance rank as competition variable.

^2^From model 2: including number of adult female groupmates as competition variable.

Table S8. Average influence of multi-year strength-consistency class (top 6 partners) in fixed time survival models, where reference class = 4, strong and consistent bonds (lowest risk class). N=83 females, 20 deaths.

| Predictor of hazard | Factor level | ß | 95% CI | | Hazard Ratio | Proportion of permutation coefficients < observed | Proportion of permutation coefficients > observed |
| --- | --- | --- | --- | --- | --- | --- | --- |
| Strength – Consistency Class  (reference class:  4, + / +  lowest risk) | 1 (- / -) | 0.43† | -1.33, 2.19 | 1.54 | | **0.985^1^, 0.987^2^** | **0.015^1^, 0.013^2^** |
|  | 2 (- /+) | 0.34† | -1.54, 2.23 | 1.41 | | 0.481^1^, 0.513^2^ | 0.519^1^, 0.487^2^ |
|  | 3 (+/ -) | 0.82† | -1.05, 2.68 | 2.26 | | **0.989^1^, 0.99^2^** | **0.011^1^, 0.01^2^** |

† Model averaged coefficient

* 95% CI does not include zero

^1^From model 1: including dominance rank as competition variable.

^2^From model 2: including number of adult female groupmates as competition variable.

Table S9. Average influence of multi-year strength-consistency class (top 6 partners) in fixed-time survival models, where reference class = 1, weak and inconsistent bonds. N=83 females, 20 deaths.

| Predictor of hazard | Factor level | ß | 95% CI | | Hazard Ratio | Proportion of permutation coefficients < observed  (more likely to decrease hazard) | Proportion of permutation coefficients > observed |
| --- | --- | --- | --- | --- | --- | --- | --- |
| Strength – Consistency Class  (reference class:  1, - / -  second to *highest* risk) | 2 (- / +) | -0.90† | -1.59, 1.42 | 0.92 | | **0.008^1^, 0.005^2^** | **0.992^1^, 0.995^2^** |
|  | 3 (+/ -) | 0.39† | -0.79, 1.56 | 1.47 | | 0.483^1^, 0.375^2^ | 0.517^1^, 0.615^2^ |
|  | 4 (+/+) | -0.43† | -2.19, 1.33 | 0.65 | | **0.015^1^, 0.013^2^** | **0.985^1^, 0.987^2^** |

† Model averaged coefficient

* 95% CI does not include zero

^1^From model 1: including dominance rank as competition variable.

^2^From model 2: including number of adult female groupmates as competition variable.
