# Supplementary material for: Stronger social bonds do not always predict greater longevity in a gregarious primate
Source: Ecol Evol. 2018 Jan 3;8(3):1604–14. doi: 10.1002/ece3.3781 (PMC5792528; doi:10.1002/ece3.3781)
Supplement: Supplementary file 8 [file ECE3-8-1604-s008.docx]

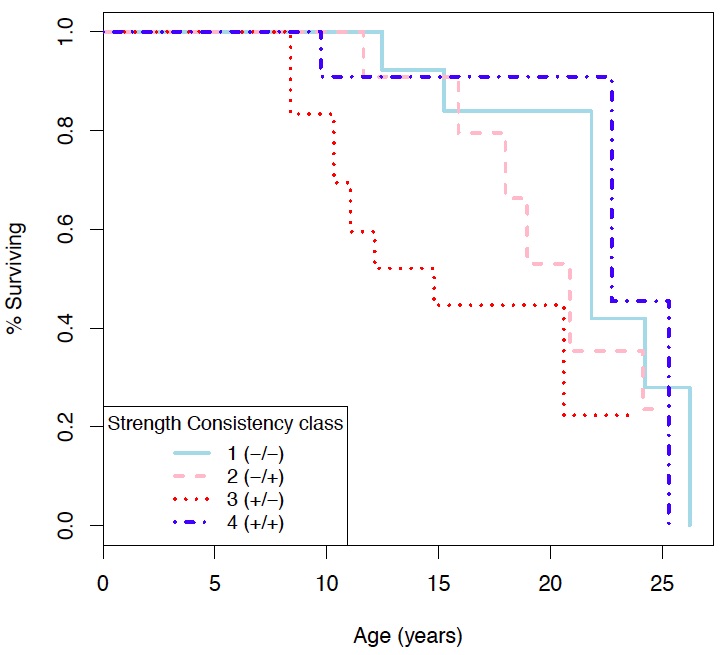


**Figure S2.** Survival curve of subjects falling into a given annual bond strength – partner consistency (with top 3 partners) class. Class 1) below average bond strength and below average partner consistency (light blue, solid line). Class 2) below average bond strength and above average consistency (pink, dashed line). Class 3) above average bond strength and below average consistency (red, small dotted line). Class 4) above average strength and above average consistency (dark blue, dashed and dotted line). Females in class 3 (+/-) have significantly lower mortality than females in class 4 (+/+)

| **Table S12.** Time dependent covariate Cox model including annual classes  of strength-consistency (st-cons) with top 3 partners, dominance rank, and number adult female groupmates. N = 354 subject-yrs. (Significant effects in bold) | | | | |
| --- | --- | --- | --- | --- |
|  | A) Base class 3 (+st/-cons) | |  |  |
|  | predictor | coef | hazard.ratio | CI |
|  | st-cons3 (1 -/-) | -1.4 | 0.25 | [-2.82,0.03] |
|  | st-cons3 (2 -/+) | -0.76 | 0.47 | [-2.01,0.48] |
|  | **st-cons3 (4 +/+)** | **-1.52** | **0.22** | **[-3.03,-0.01]** |
|  | Rank | 0.16 | 1.17 | [-0.32,0.64] |
|  | af.groupmates | -0.13 | 0.88 | [-0.6,0.34] |
|  |  |  |  |  |
|  |  |  |  |  |
|  | B) Base class 4 (+st/+cons) | |  |  |
|  | predictor | coef | hazard.ratio | CI |
|  | st-cons3 (1 -/-) | 0.12 | 1.13 | [-1.44,1.69] |
|  | st-cons3 (2 -/+) | 0.76 | 2.13 | [-0.69,2.2] |
|  | **st-cons3 (3 +/-)** | **1.52** | **4.57** | **[0.01,3.03]** |
|  | Rank | 0.16 | 1.17 | [-0.32,0.64] |
|  | af.groupmates | -0.13 | 0.88 | [-0.6,0.34] |
|  |  |  |  |  |
|  |  |  |  |  |
|  | C) Base class 1 (-st/-cons) | |  |  |
|  | predictor | coef | hazard.ratio | CI |
|  | st-cons3 (2 -/+) | 0.63 | 1.88 | [-0.73,1.99] |
|  | st-cons3 (3 +/-) | 1.4 | 4.04 | [-0.03,2.82] |
|  | st-cons3 (4 +/+) | -0.12 | 0.88 | [-1.69,1.44] |
|  | rank | 0.16 | 1.17 | [-0.32,0.64] |
|  | af.groupmates | -0.13 | 0.88 | [-0.6,0.34] |

| **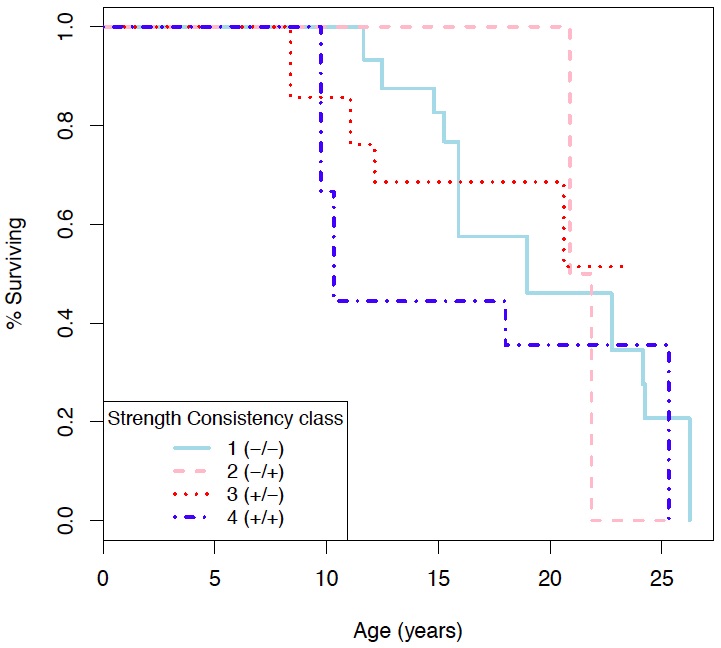**  **Figure S3.** Survival curve of subjects falling into a given annual bond strength – partner consistency (with top 6 partners) class. Class 1) below average bond strength and below average partner consistency (light blue, solid line). Class 2) below average bond strength and above average consistency (pink, dashed line). Class 3) above average bond strength and below average consistency (red, small dotted line). Class 4) above average strength and above average consistency (dark blue, dashed and dotted line). Female survival did not vary by class.  **Table S13.** Time dependent covariate Cox model including annual classes of strength-consistency (st-cons) with top 6 partners, dominance rank, and number adult female groupmates. N = 354 subject-yrs | | | | |
| --- | --- | --- | --- | --- |
|  | A) Base class 3 (+st/-cons) | |  |  |
|  | predictor | coef | hazard.ratio | CI |
|  | st-cons6 (1 -/-) | 0.04 | 1.04 | [-1.19,1.27] |
|  | st-cons6 (2 -/+) | -0.58 | 0.56 | [-2.42,1.26] |
|  | st-cons6 (4 +/+) | -0.02 | 0.98 | [-1.55,1.52] |
|  | rank | 0.1 | 1.1 | [-0.39,0.58] |
|  | af.groupmates | -0.29 | 0.75 | [-0.78,0.21] |
|  |  |  |  |  |
|  |  |  |  |  |
|  | B) Base class 4 (+st/+cons) | |  |  |
|  | predictor | coef | hazard.ratio | CI |
|  | st-cons6 (1 -/-) | 0.05 | 1.06 | [-1.31,1.42] |
|  | st-cons6 (2 -/+) | -0.56 | 0.57 | [-2.32,1.19] |
|  | st-cons6 (3 +/-) | 0.02 | 1.02 | [-1.52,1.55] |
|  | rank | 0.1 | 1.1 | [-0.39,0.58] |
|  | af.groupmates | -0.29 | 0.75 | [-0.78,0.21] |
|  |  |  |  |  |
|  |  |  |  |  |
|  | C) Base class 1 (-st/-cons) | |  |  |
|  | predictor | coef | hazard.ratio | CI |
|  | st-cons6 (2 -/+) | -0.62 | 0.54 | [-2.32,1.09] |
|  | st-cons6 (3 +/-) | -0.04 | 0.96 | [-1.27,1.19] |
|  | st-cons6 (4 +/+) | -0.05 | 0.95 | [-1.42,1.31] |
|  | rank | 0.1 | 1.1 | [-0.39,0.58] |
|  | af.groupmates | -0.29 | 0.75 | [-0.78,0.21] |
